# Development of a radio thin-layer chromatography scanner: Design and qualification

**DOI:** 10.1186/s41181-025-00413-z

**Published:** 2025-12-19

**Authors:** Tomás Chivato Martín-Falquina, Elena Miñana Olmo, Ángela Alonso García, Matthias Rosezky

**Affiliations:** 1 Unidad de Radiofarmacia, Hospital General Universitario Santa Lucía, c/ Minarete s/n, 30202 Cartagena, Spain; 2 NuclearPhoenix, Vienna, Austria

**Keywords:** Thin layer chromatography, RadioTLC, Radiochemical purity, Silicon photomultiplier

## Abstract

**Background:**

Radiochemical Purity (RCP) assessment is a fundamental quality control parameter in radiopharmaceutical production. Thin-Layer Chromatography (TLC) using a radio-TLC scanner is the most common method employed to determine RCP. However, commercial devices are often expensive and may not be accessible for research environments or budget-constrained laboratories. This study presents the development of a low-cost, open-source radio-TLC scanner utilizing a silicon photomultiplier-based scintillation detector and a linear actuator. The system was designed to scan TLC strips and provide quantitative analysis. Validation for analysis of technetium-99m labeled compounds followed regulatory guidelines and included assessment of background noise, linearity, repeatability, positional accuracy, and comparative analysis against a commercial scanner.

**Results:**

The system demonstrated good analytical performance, and comparative testing revealed a strong agreement with the results obtained using a commercial radio-TLC scanner.

**Conclusion:**

The custom radio-TLC scanner represents a viable and affordable alternative for radiochemical purity assessment in radiopharmaceutical quality control while maintaining compliance with good manufacturing practice standards.

## Background

The preparation and quality control of radiopharmaceuticals require strict adherence to Good Manufacturing Practices, whether carried out in an industrial or small-scale setting (U.S. Food and Drug Administration 2024; United States Pharmacopeia and National Formulary 2021; European Commission: Eudralex - Volume 4 2008; Gillings et al. 2021; Pharmaceutical Inspection Co-operation Scheme 2014; World Health Organization 2020). One of the primary quality control tests to finished radiopharmaceutical products is the assessment of Radiochemical Purity (RCP), the analytical procedure used to determine the proportion of total radioactivity in a radiopharmaceutical preparation present in the desired chemical form (Kowalsky and Weatherman^2021^). This test quantifies the presence of radiochemical impurities, which may arise during synthesis, labeling, or storage, and decrease the overall quality of both diagnostic and therapeutic radiopharmaceuticals, causing unnecessary radiation exposure to patients. RCP testing thus ensures that the radiopharmaceutical meets its predefined specifications for clinical or research use. When performed by Thin-Layer Chromatography (TLC), the test involves applying a drop of the radiopharmaceutical onto chromatographic strips, developing them with an appropriate mobile phase, and then analyzing the distribution of radioactivity along the strip using a radioactivity scanner. The resulting chromatogram allows for the identification and quantification of the radiolabeled compound and its potential impurities based on their respective Retention Factor (Rf). Radio-TLC scanners are widely used in radiopharmaceutical laboratories. They include a collimated detector coupled to a ratemeter that outputs the signal along the strip to a chart recorder. Linear analyzers, a similar device, use gas proportional counters and a grid of electrodes to simultaneously count the activity of the strip in a set number of channels with high sensitivity (Theobald 2010). However, these scanners’ high cost limits accessibility for research groups and smaller facilities, which have to resort to cutting the strips and counting the activity in a dose calibrator or scintillation counter, autoradiography or gamma camera scanning (Zolle 2007). These methods are slower, have higher variability and require more manipulation, leading to higher contamination risks (Ballinger and Blower 2011).

Advances in solid-state radiation detection and microcontrollers provide an opportunity to develop a cost-effective alternative. Silicon Photomultipliers (SiPMs) are a relatively new technology in photon detection, offering several advantages over traditional Photomultiplier Tubes (PMTs). They provide a compact, robust, and efficient alternative for applications requiring high sensitivity and fast response times, such as medical applications and particle detection (Wagatsuma et al. 2017; Hahn et al. 2024; Tsutsui et al. 2020; Recker et al. 2019). Unlike PMTs, which require fragile vacuum tubes, high-voltage operation, and can be influenced by magnetic fields, SiPMs are solid-state semiconductor devices that operate at lower voltages, reducing power consumption (Onsemi 2023). Additionally, a number of studies indicate that, in typical use cases, SiPMs exhibit higher photon detection efficiency than traditional PMTs, although acquiring definitive figures for general applications has proven to be difficult (Li et al. 2023; Kovaltchouk et al. 2005; Tsutsui et al. 2020; Wagatsuma et al. 2017; Gundacker and Heering 2020). Higher photon detection efficiency allows for improved sensitivity of the sensor and a good uniformity of response. In recent years, programmable microcontroller boards such as Raspberry Pi and Arduino have revolutionized prototyping across multiple fields. These platforms provide a versatile and accessible solution for developing custom electronic systems, enabling researchers and engineers to build functional prototypes with minimal financial and technical barriers. One of the key advantages of these boards is their flexibility and modularity. They can interface with a wide range of sensors, actuators, and communication modules, making them suitable for applications in automation, data acquisition or environmental monitoring (Oellermann et al. 2022; Kondaveeti et al. 2021; Mathe et al. 2024). Their open-source nature provides a collaborative ecosystem where developers can access extensive documentation, libraries, and community-driven support, accelerating the development process.

This study presents the design and implementation of a radio-TLC scanner using a SiPM detector, a Raspberry Pi Pico microcontroller, and a linear actuator system. The radio-TLC scanner was qualified for the analysis of technetium-99 m radiopharmaceuticals, performing real-time chromatogram acquisition and data analysis while ensuring compliance with regulatory standards.

## Materials and Methods

**Fig. 1:**
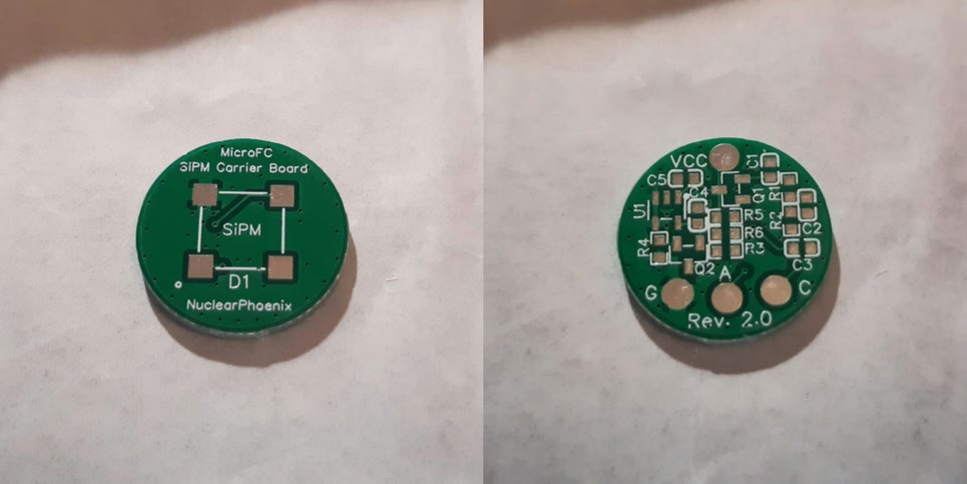
Images of the MicroFC SiPM Carrier Board, taken from Rosezky 2025a. Left: front side where the SiPM will be mounted. Right: back side with the support circuitry and electrical connections. The diameter of the board is 18 mm, enabling a compact build size

**Fig. 2:**
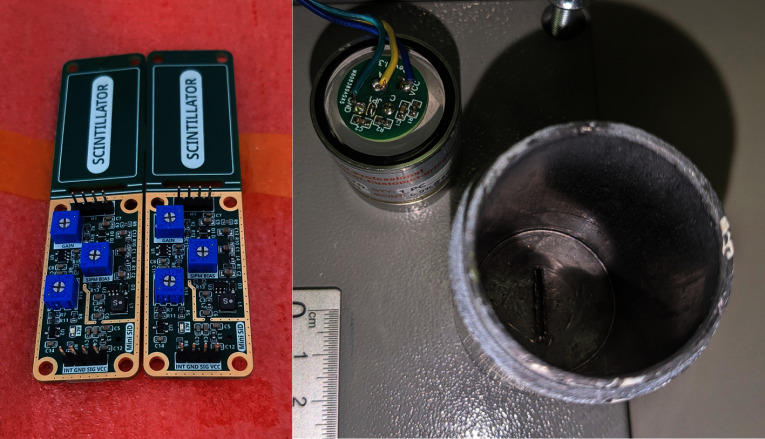
Left: Image of two Mini SiPM Driver boards with focus on the electronics area in the front. Right: Wired SiPM Carrier Board on top of the exposed side of the scintillation crystal. After completely wrapping it with insulating tape, the assembly was secured inside the lead shield next to it

The detector was based on a solid scintillator, consisting of an encapsulated cylindrical NaI(Tl) crystal (Epic-Crystal, China) with dimensions of 25.4 mm in diameter and 25.4 mm in height. A SiPM (MICROFC-60035-SMT-TR. Onsemi, USA) (Onsemi 2018, 2022) was soldered onto a MicroFC SiPM Carrier Board (Fig. 1) (Rosezky 2025a). This board was specifically designed for the 6 mm MicroFC SiPM used here and has the goal of housing the sensor mechanically on the one side of the PCB with additional support circuitry to improve its performance on the opposite side. The circuitry consists of the SiPM bias filtering, as well as a simple temperature compensation circuit to prevent changes in SiPM gain if the temperature varies (Kuznetsov 2018). Since the SiPM and other circuitry do not produce any significant waste heat, this is mostly related to the ambient temperatures and critical in scenarios where temperature stability can not be guaranteed outside of a few degrees. In this application, three connections to the carrier board were made: the SiPM bias input, ground, and the SiPM photocurrent output. The crystal was optically coupled to the SiPM using silicone grease, wrapped in white insulating tape, and housed in a lead vial shield (internal diameter: 34 mm, lead thickness: 2 mm) with a 20 x 1 mm slit acting as a collimator (Fig. 2).

**Fig. 3:**
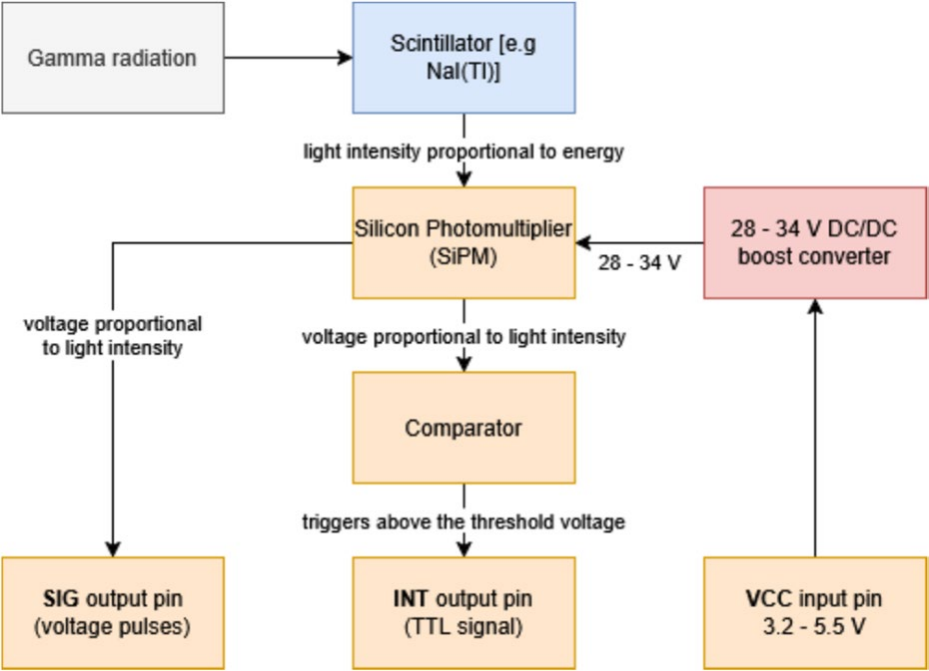
Working principle of the Mini-SiD with a simple scintillator assembly, taken from Rosezky 2025b

To handle the signals coming from the SiPM and to produce the necessary biasing voltage for the sensor, a special processing board is needed. The Mini SiPM Driver board (*Mini-SiD*, figure 2), is a small development board designed to be embedded into other projects and offers an integrated power supply for the SiPM within a range of 30 V, compact size (50 $\times$ 25 mm area for the electronics), and low power consumption. Most importantly, though, it houses simple pulse processing and a discriminator with a digital pulse output corresponding to the signal coming from the SiPM (Rosezky 2025b). The signal gain and discriminator threshold can be adjusted to the project’s needs. The working principle of the device is shown in fig. 3. The digital pulse output from the Mini-SiD can then be used to easily count the pulses of light detected by the SiPM in any time interval. This is done with a powerful-enough microcontroller that is able to register the short digital pulses and do further information processing. The Mini-SiD itself is in turn powered by the microcontroller via a single 3.3 V pin.

A Raspberry Pi Pico, a compact and cost-effective programmable board, was used as microcontroller. It includes a dual-core ARM Cortex processor, 264 kB of SRAM, and 2 MB of onboard Flash memory. The board offers 26 multifunction GPIO pins and supports standard interfaces such as I2C, SPI, UART, PWM, and a 12-bit ADC. With its low power consumption and support for programming in MicroPython, the Raspberry Pi Pico was deemed appropriate for the control tasks.

**Fig. 4:**
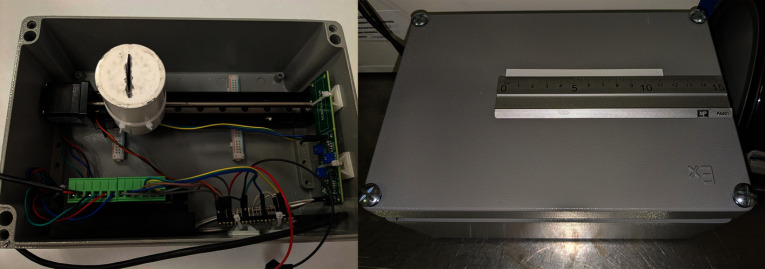
Left: Internal layout of the device components. The white cylinder is the collimator that encloses the detector moving along the screw actuated by the stepper motor. On the right wall rests the Mini-SiD board. On the bottom one, the motor driver and the Raspberry Pi Pico. Right: Device in operation with a TLC strip in place

The linear actuator consisted of a standard four-wire NEMA 17 stepper motor coupled to a sliding platform with a 150 mm stroke and 2 mm lead screw pitch, moving in 0.5 mm steps. The motor was controlled by a TB6600 driver in $\frac{1}{2}$ microstepping mode using a 12 V, 2 A power supply. A limit switch defined the scan’s starting position. Although the effective scanning range was 127 mm, software limited it to 120 mm for safety. The detector, electronic boards, and actuator were mounted within an aluminum enclosure ($240 \times 160 \times 100\,\hbox {mm}$) (Fig. 4), with a 150 mm ruler attached to the top to facilitate positioning of chromatographic strips. To perform a scan, the user puts the developed strip in the designated area on top of the radio-TLC scanner and launches the acquisition from a PC connected via USB. The radio-TLC scanner itself has no onboard controls or user interface elements other than a switch to manually interrupt a running scan.

**Fig. 5:**
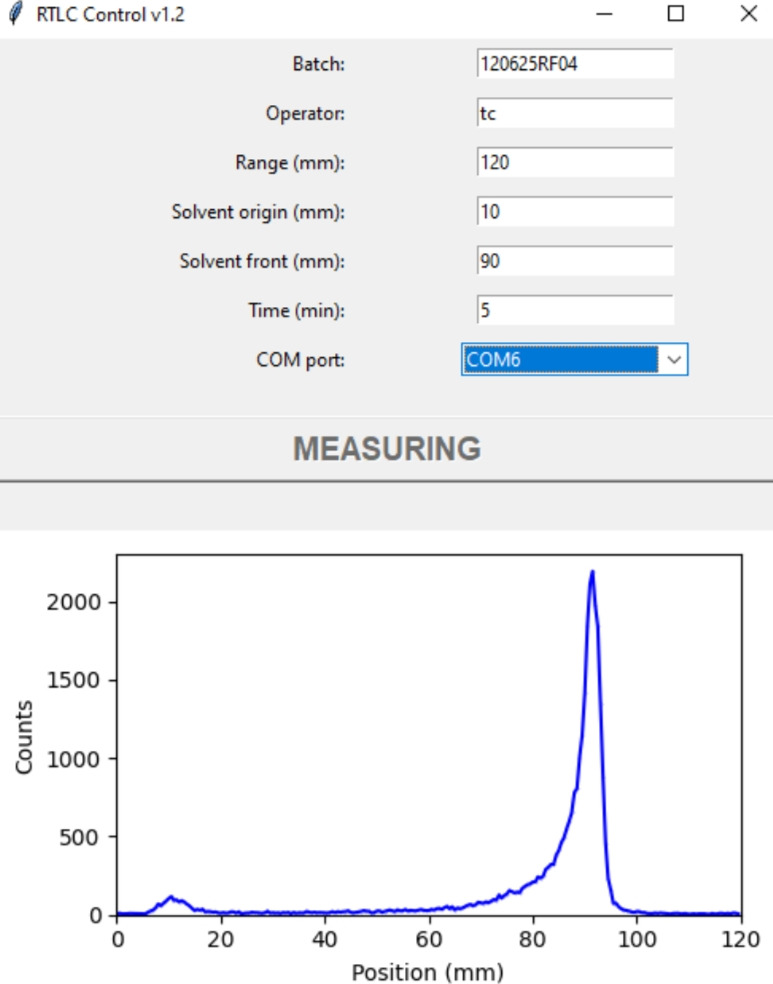
Acquisition as seen in the control software during the scan of a $5\,\upmu \hbox {l}$ sample of [^99 m^Tc]Tc-HMPAO developed with methyl ethyl ketone

Three dedicated programs were developed for system operation: a Graphical User Interface (GUI), a firmware, and a report generator (Chivato Martín-Falquina 2025). The GUI, designed in Python 3, allowed the user to input the measurement parameters, including batch number, operator, scan range, acquisition time, and solvent origin and front values. Once the operator starts the acquisition, this information is sent to the microcontroller, which returns the counting data for each position in real time to the PC, which generates a live chromatogram display (Fig. 5). Upon completion of the acquisition, the chromatogram and all relevant metadata are saved in a Comma-Separated Value (CSV) file. Finally, a SHA-256 hash is calculated and added to the CSV as a footprint (National Institute of Standards and Technology 2015). The microcontroller firmware was programmed using a MicroPython script performing four key functions: receiving measurement parameters from the PC, controlling the detector movement, counting pulses from the Mini-SiD board and transmitting the data back to the PC. For data analysis and reporting, a Microsoft Excel spreadsheet was programmed. Upon loading the CSV file, the chromatogram is displayed, and the user manually enters region identifiers and integration bounds for the peaks and background. The spreadsheet then calculates for each region the peak area and relative percentage after background subtraction, retention factor as ratio of the distance traveled by the solute to the distance traveled by the solvent front, Signal-to-Noise Ratio (SNR) as twice the peak height divided by the baseline noise (United States Pharmacopeia and National Formulary 2023; European Directorate for the Quality of Medicines & HealthCare 2023), and sharpness as Full Width at Half Maximum (FWHM). Several measures were adopted to adhere to Good Practices for Computerized Systems (Pharmaceutical Inspection Co-operation Scheme 2007). The GUI only allows for the execution of acquisitions, and any values entered by the user in each field are validated by the software itself. The integration spreadsheet is password-protected to prevent users from unauthorized modifications, while changes made by the administrator are recorded in the audit trail. To guarantee the integrity of raw data, the spreadsheet verifies the SHA-256 hash to ensure that the CSV data has not been altered. Once the data integration process is complete, the user can validate the results and archive the report using an electronic signature. Access to the PC itself was restricted to approved users only, and its contents routinely backed-up. The system was designed as a plug-and-play device. The PC software was compiled into a standalone executable file that requires only a 64-bit Microsoft Windows system with an available USB port.

To qualify the radio-TLC scanner, a series of tests were performed following international guidelines (International Council for Harmonisation 2024; United States Pharmacopeia and National Formulary 2023, 2023, 2023; European Commission: Eudralex - Volume 4 2015; European Directorate for the Quality of Medicines & HealthCare 2023, 2023, 2018; Pharmaceutical Inspection Co-operation Scheme 2007; Todde et al. 2017; Gillings et al. 2020). Background activity was assessed via triplicate 5-min acquisitions, and the average count rate was calculated. For linearity, a $5\,\upmu \hbox {L}$ sample containing 18.5 MBq of technetium-99 m was applied to the center of an instant TLC strip. The strip was then fixed with adhesive tape, and scanned nine times over three days as the sample decayed to 2.5 kBq. Measured count rates were plotted against the calculated activity to assess response linearity (target: R $\ge$ 0.99). Repeatability was evaluated by scanning six consecutive times for 2 min a strip spotted with 7.4 MBq of technetium-99 m on one end and 0.4 MBq on the other—simulating a 5% impurity—and calculating the coefficient of variation of the impurity values obtained (target: CV $\le$ 5%). The Limit of Quantification (LOQ) was defined as the lowest activity yielding a peak with a SNR $\ge$ 10. The working range of the instrument was established from the highest activity verified in the linearity test to 200 times the LOQ, to guarantee the capability to quantify impurities down to 0.5% of the lower limit. To facilitate comparison with the activity concentrations typically found in technetium-99 m labeled kits—which are prepared as liquid samples of known volume—the LOQ and the working range were expressed as activity concentration—derived from the $5\,\upmu \hbox {l}$ sample volume—rather than absolute activity. Positioning accuracy was evaluated by spotting a 10 cm strip with nine $5\,\,\upmu \hbox {L}$ technetium-99 m samples spaced 1 cm apart, and comparing the calculated and observed retention factors. To assess agreement, the device was benchmarked against a qualified commercial instrument used as reference system (MiniGita. Elysia-Raytest, Germany). In this scanner, the strip is placed on top of a sliding table that passes under a collimated Bismuth Germanium Oxide (BGO) crystal based scintillation detector with a traditional photomultiplier. RCP tests (n = 100) were conducted in parallel on both systems using 120 mm instant TLC strips and $5\,\upmu \hbox {L}$ samples of technetium-99 m labelled radiopharmaceuticals, covering a variety of compounds including oxidronate, exametazime, mertiatide, octreotide, succimer, MIBI and albumin nanocolloid. The last three correspond to single-strip assays, whereas the remaining used dual-strip systems in which the overall RCP value was used for method comparison. Each strip was developed according to the respective kit manufacturer’s instructions (HDP 2020; Exametazime 2017; Nephromag 2016; Tektrotyd 2020; Renocis 2025; Stamicis 2021; Nanocolloidal human albumin 2020), scanned for 2 min in each device and manually integrated to calculate the RCP. Method correlation was evaluated using Pearson’s correlation coefficient (target: R $\ge$ 0.95), and agreement was further analyzed using Bland–Altman plots to assess bias and Limits of Agreement (LOA) (Altman and Bland 1983; Bland and Altman 1999). Normality in the distribution of the differences between the two methods was verified using the Shapiro-Wilk test. LOA were defined as mean difference $\pm 1.96$ times the standard deviation.

## Results

The radio-TLC scanner showed satisfactory performance, rapid operation, and high sensitivity, although the recorded count rates were approximately ten times lower than those of the reference system. Stepping in 0.5 mm intervals equaled to 240 acquisition channels over a 120 mm strip, resulting in high-quality chromatograms with good resolution, as indicated by FWHM values as low as 3.5 mm, similar to the ones obtained on the reference system during the comparison tests (Fig. 7). The results of the qualification tests are summarized in Table 1.

**Table 1:** Summary of qualification results

Parameter	Acceptance criteria	Result
Background	–	0.31 ± 0.04 cps
Linearity	R > 0.99	R = 0.9996
Repeatability	CV < 5%	CV = 2.6%
Limit of quantification	SNR $\ge$ 10	0.5 MBq/ml (SNR = 10)
Range	–	3700–100 MBq/ml
Position accuracy	Deviation from calculated RF $\le$ 10%	Pass

**Fig. 6:**
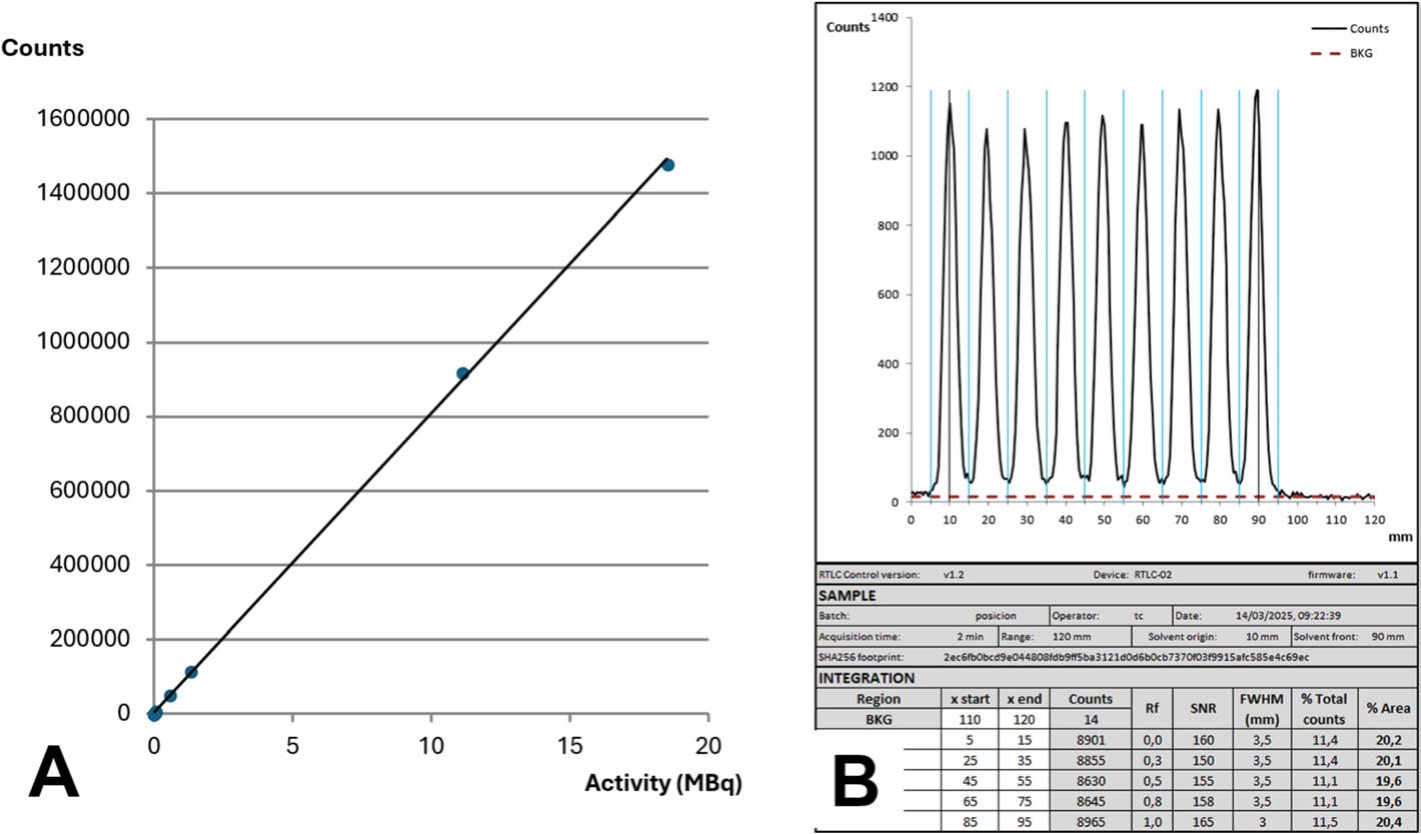
**A** Linearity test plot. **B** Position accuracy results. The peaks not integrated—corresponding to Rf of 0.1, 0.4, 0.6 and 0.9—also showed no deviation

Background noise was found to be low, allowing for stable baselines in the chromatograms. The detector had a good linear response and repeatability. The qualified working range covers technetium-99m activity concentrations most frequently used in hospital radiopharmacies, the most common one in our case being 1480 MBq/ml. The linear actuator was able to accurately move the detector, as no difference was found between the activity spot place and the position at which they were detected (Fig. 6). The comparative analysis against the reference system revealed a strong correlation (R = 0.99). The Bland-Altman plot showed a mean difference (bias) of $-$0.12 ± 0.21%, with most data points clustered closely around the mean and within a narrow range of differences ($-$0.54–0.3%) (Fig. 8). No trend was observed in the bias across RCP values, with the differences showing a normal distribution throughout the tested range (p > 0.05). The bias was more pronounced in double-strip tests, which accumulated differences from two independent measurements. All the outliers beyond the LOA corresponded to double-strip tests.

**Fig. 7:**
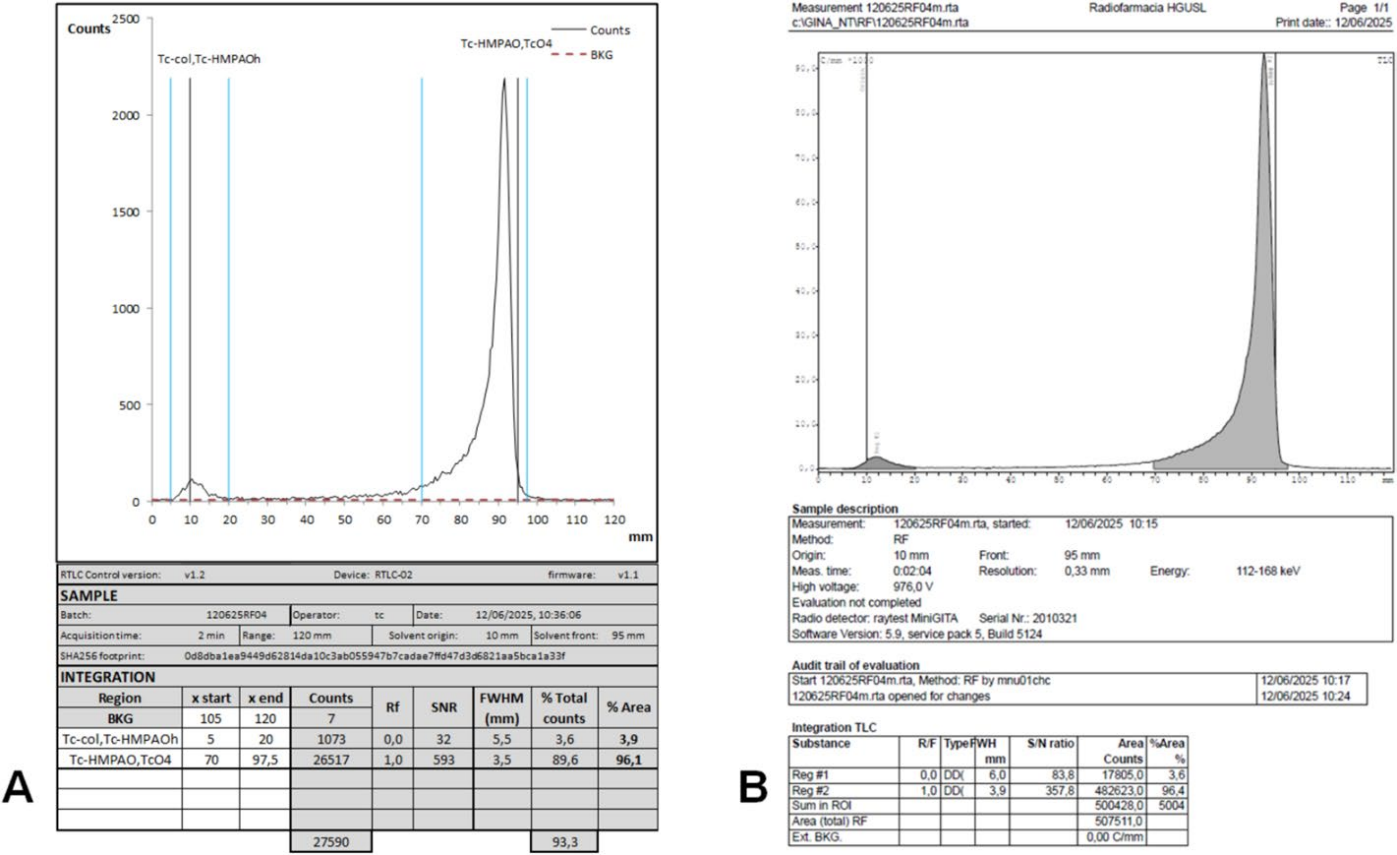
**A** Spreadsheet integration form for the sample in Fig. 5. Only the content of white background cells can be modified. Sample data is automatically loaded from the CSV file. **B** Report of the same strip read on the Minigita

**Fig. 8:**
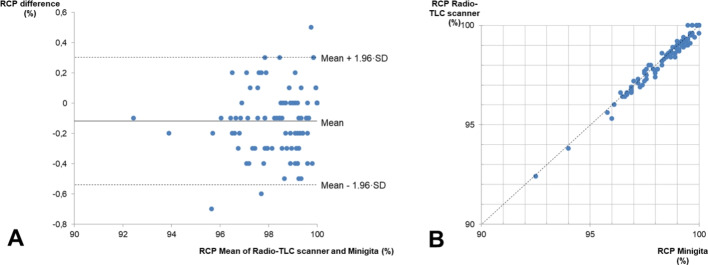
Comparative analysis between the Radio-TLC scanner and the Minigita. **A** Bland-Altman Plot. **B** Results correlation

## Discussion

Since the 1960 s, several in-house solutions for strip scanning have been reported, preceding the widespread adoption of commercial radio-TLC scanners (Rosenberg and Bolgar 1963; Sunderland 1975; Williams et al. 1977; Jansholt et al. 1980; Harris et al. 1984). While these devices performed well, their reproduction today would be challenging due to outdated components and designs. More recently, Jeon et al. adapted a scintillation crystal array coupled with a photodiode array, showing comparable ROI counts to a Bioscan AR-2000, but with poorer resolution (Jeon et al. 2017). Ekoume et al. developed a method to assess RCP using only a survey meter with good performance, but as it involved cut and count, it lacked spatial resolution and was more labor-intensive (Ekoume et al. 2020). In the last few years, Cerenkov luminescence imaging has been used to perform RCP determination, resulting in systems with high sensitivity and resolution, capable of processing larger amounts of samples and able to also spot artifacts in the plates (Dam and Chatziioannou 2021; Wang et al. 2020; Webb et al. 2024). However, this method requires specialized equipment and software.

The objective of our study was achieved, as the custom radio-TLC scanner resulted in an efficient, compact, and easy-to-use device. Assembly did not require specialized tools, and the Mini-SiD and SiPM carrier boards were sourced from a commercial PCB manufacturer (PCBWay, China). However, reproducing the system demands an intermediate level of technical proficiency. Successful construction and operation of the scanner require a basic understanding of electronics—such as connecting sensors, switches and driver boards—as well as the ability to handle simple soldering tasks and read circuit diagrams. Users should also be familiar with microcontroller programming and computer interfacing, to upload firmware, configure acquisition parameters, and understand data transfer between the device and the PC. Basic mechanical assembly skills are necessary to align the actuator, mount the detector, and ensure stable operation of moving components using standard tools. Furthermore, knowledge of quality control principles and chromatographic techniques is essential to correctly perform the qualification and interpret analytical results. The total time required to set up the device for the first time was approximately six weeks, including material procurement, assembly, and qualification. The general efficiency and range of the detector was satisfactory and similar to the device described by (Jansholt et al. 1980), which had a 1.85 kBq lower range limit. The radio-TLC scanner yielded results that closely matched the reference system, as was expected since both methods operate on the same fundamental principle. The precision of both instruments—expressed as coefficient of variation—is under 3%, so the minor differences observed in the RCP values, all below 1%, may be attributed to the inherent variability of the methods. Since the bias obtained in the comparative study was small, and given that the typical threshold for impurities in technetium-99 m radiopharmaceuticals generally ranges from 2% to 5% (Kowalsky and Weatherman 2021), both methods could be considered interchangeable, although the systematic difference should be accounted for.

Potential hardware improvements to the project could be introduced, such as using a larger screw to allow longer scanning distances or adapting it to determine the energy spectrum using a board designed for this purpose (Rosezky 2025c; Bonifacio et al. 2025). The moving stage might be upgraded to a more advanced solution with an encoder for better precision and position control. However, in our experience the performance of the stepper motor and limit switch was sufficiently accurate, and the added complexity would not offer a meaningful advantage. Mounting the sliding table on top of another linear actuator moving perpendicularly would allow for automatic multistrip scanning. Finally, radiopharmaceuticals labeled with higher-energy radioisotopes could be analyzed by adding a thicker collimator. On the software side, automatic detection and integration of peaks may be implemented in the spreadsheet. The PC interface could theoretically have also been used for data integration, uniting all software. However, we chose to perform the integration as a separate step in order to facilitate potential modifications to the integration spreadsheet, without compromising the stability or functionality of the control software. Furthermore, the Excel-based integration sheet may be replaced by an alternative solution developed in RStudio, LabVIEW, or a similar programming environment.

The total cost of materials and building was USD 250. The manpower required for assembly and testing was not included in this estimation. While the project is designed to be accessible and is thoroughly documented (Rosezky 2025a, 2025b; Chivato Martín-Falquina 2025), users without prior technical experience may benefit from the collaboration of a technician, or an engineering or physics student to facilitate initial setup. Alternatively, outsourcing the construction to a specialized company could be considered; however, the associated cost could be significant, possibly defeating the purpose of the device. Maintenance costs are expected to be also low due to the general simplicity of the system, and any malfunction can be resolved simply by replacing the affected component, as they are all inexpensive. In comparison, commercial radiochromatography systems such as Minigita, Scan-Ram (LabLogic, United Kingdom), TLC-204 (Comecer, Italy), or Bioscan AR-2000 (Eckert & Ziegler, Germany) represent—as of 2025—an investment ranging from approximately USD 25,000 to 40,000 (excluding taxes), depending on the equipment configuration, software licenses, and other specifications. In addition, this cost does not account for preventive and corrective maintenance services. Radiopharmacies in developing countries or ones that serve small or low-volume nuclear medicine services often face significant economic constraints that limit their operational capacity (Adedapo et al. 2013; Dondi et al. 2011; Cutler et al. 2021). The high costs associated with equipment, maintenance, and facility requirements can restrict activities to basic kit reconstitution or small-scale synthesis, using simplified methods and instrumentation. As a result, there is considerable heterogeneity in quality control practices, with many sites relying on techniques such as cut-and-count instead of standardized approaches like validated radio-TLC systems. According to the operational classifications of the IAEA, these facilities typically operate at level 1 or 2, with limited ability to perform advanced production, full quality control testing, or research (International Atomic Energy Agency 2008). These limitations underline the need to ensure the safe and consistent use of radiopharmaceuticals across diverse clinical settings.

Our device has several drawbacks. It requires an extensive validation effort and comes without technical support, in contrast to a commercial alternative with an after-sales service available. The position of the detector inside the case makes it difficult to change the collimator to measure higher energy isotopes, and introduces more distance between detector and strip, leading to lower count rates. Nevertheless, scanning times of 2 min are enough for most tests, while decayed or diluted samples with radioactive concentration under 370 MBq/ml might require up to 5 min or spotting larger volumes onto the strip. Software validation is a particularly complex task that must be carefully addressed, especially considering the challenges related to data integrity and user control. The correct functioning of the Radio-TLC scanner should be verified at defined intervals (Gómez-Perales et al. 2016; International Atomic Energy Agency 2018), and the device should follow the requalification policies established by each institution to ensure that it remains in a controlled state throughout its life cycle.

## Conclusions

Our study presents the development and qualification of a low-cost, open-source radio-TLC scanner suitable for RCP assessment of technetium-99 m radiopharmaceuticals. The system demonstrated reliable performance, and the qualification results confirm that it can serve as a viable alternative to expensive commercial systems, particularly in resource-limited settings or as a backup in established radiopharmacies. Its modular design, minimal assembly requirements, and open-source software make it accessible for reproduction and adaptation in any radiopharmacy unit.

## Data Availability

Not applicable.

## Abbreviations

BGO: Bismuth germanium oxide
CSV: Comma-separated value
FWHM: Full width at half maximum
GUI: Graphical user interface
LOA: Limits of agreement
LOQ: Limit of quantification
PMT: Photomultiplier tube
RCP: Radiochemical purity
Rf: Retention factor
SiPM: Silicon photomultiplier
SNR: Signal-to-noise ratio
TLC: Thin-layer chromatography
